# 

**Published:** 1893-12-09

**Authors:** 


THE HOSPITAL.

PRICE TWOPENCE.

No. 376, Vol. XV.—Dec. 9th, 1893.

[Registered at the General Post Office as a Newspaper for transmission in the United Kingdom and Abroad.]

DECEMBER 9TH,

WITH EXTRA NURSING SUPPLEME

Per Annum, 8s. 6d., post free.

Monthly Parts, 6d.; post free, 8d.

Ditto per Annum, 8s., post free.

### THE HOSPITAL

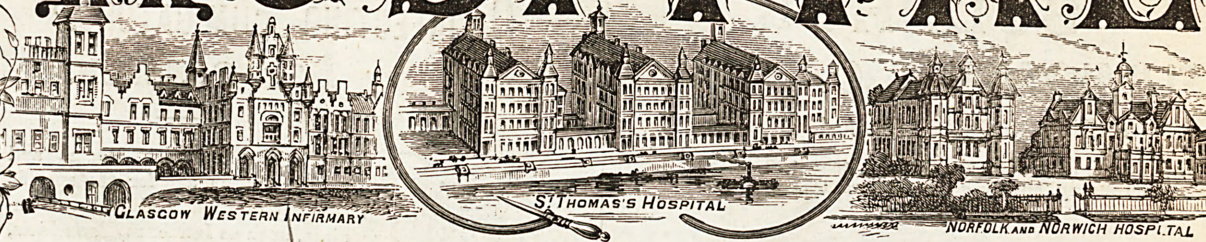

WITH EXTRA NURSING SUPPLEMENT.

No. 376. Vol. XV. SATURDAY, DECEMBER 9TH, 1893. [TWOPENCE.

